# Locally weighted PCA regression to recover missing markers in human motion data

**DOI:** 10.1371/journal.pone.0272407

**Published:** 2022-08-08

**Authors:** Hai Dang Kieu, Hongchuan Yu, Zhuorong Li, Jian Jun Zhang

**Affiliations:** 1 University of Engineering and Technology, Vietnam National University, Hanoi, Vietnam; 2 National Centre for Computer Animation, Bournemouth University, Poole, United Kingdom; 3 School of Computer Science, Zhejiang University City College, Hangzhou, China; Institut de Robotica i Informatica Industrial, SPAIN

## Abstract

“Missing markers problem”, that is, missing markers during a motion capture session, has been raised for many years in Motion Capture field. We propose the locally weighted principal component analysis (PCA) regression method to deal with this challenge. The main merit is to introduce the sparsity of observation datasets through the multivariate tapering approach into traditional least square methods and develop it into a new kind of least square methods with the sparsity constraints. To the best of our knowledge, it is the first least square method with the sparsity constraints. Our experiments show that the proposed regression method can reach high estimation accuracy and has a good numerical stability.

## Introduction

Motion Capture (MoCap) technology is widely applied to our daily life, ranging from clinical purposes, sport coaching to movie visual effect production, computer animation [1–4] and VR/AR such as the iPad’s LiDAR sensor. We aim at one kind of MoCap data, i.e. 3D skeletal motion data, since some of usual problems (e.g. missing markers or occlusion, short-duration high frequency noise or jitter) always result in gaps in datasets, which is called as the “Missing Marker Problem” [5]. Although there are some commercial software available which can provide powerful tools for aiding in the cleanup of MoCap data [6], it can still often take several hours per capture and is almost always the most expensive and time consuming part of the pipeline. A rising challenge is to improve the accuracy of recovering gaps and the computational efficiency for evergrowing data [7]. There have been a certain number of presented measures to address this problem. Traditional approaches [8–10], utilizing the linear interpolation, spline interpolation, monotone piecewise cubic interpolation as well as Kalman filter, can successfully recovery gaps. Resorting to the available temporal information, they can work in real-time. However, these methods usually rely on the continuity of motion sequences. Manual intervention is still required when markers are missing for a long period of time, or missing from the very beginning [11, 12]. Thus this kind of methods may be unsuitable for long duration of missing joints [13, 14].

Besides, several methods, Li et al. [15, 16] and Tan et al. [17], employed the Linear Dynamic System (LDS) technology and successfully applied it to real-time applications. But they failed in a large ratio of occluded markers [18]. Moreover, Singular Value Thresholding (SVT) [19] and Non-negative Matrix Factorization (NMF) [11] approaches were employed as well in [12, 20, 21]. The distinct advantage is to take charge of the sparsest approximation to redundant motion datasets. The further research [22] aimed to clean up motion data through the low-rank matrix decomposition technology. However, such low rank approximation methods usually require prior knowledge of skeleton constraints, or the availability of a prerecorded dataset to recalculate skeleton constraints. Unfortunately they still led to unrealistic recovery [23], particularly the multiple missing markers’ scenarios [5, 24]. [18] further shows that when joints move up and down sharply and radically in motion sequences, all methods have to suffer too many “outliers” in the context of interpolation. The numerical stability of algorithms should be given priority. Apart from that, Federol [5, 24] employed the principal component analysis (PCA) approach to the multiple missing markers’ scenarios. The improvement is limited since they didn’t utilize training datasets. To take advantage of training datasets, Liu et al. [25] proposed a method of combining PCA and K-mean clustering. Our previous work [26] also made an attempt to tackle this issue. However, the numerical stability of algorithms is still a major challenge.

The main contribution of this paper is to introduce the multivariate tapering approach [27] to traditional least square methods and further develop it into the locally weighted PCA regression method for the “missing marker problem”. To the best of our knowledge, it is the first least square method with the sparsity constraints. Essentially, thanks to a sparse approximate covariance, it effectively suppresses the errors from redundant observation data and drastically improves the accuracy of estimations. The traditional least square methods just cannot handle the redundancy of input data well. Our experiments validate that the proposed locally weighted PCA regression method has a good numerical stability.

## Methods

Our basic idea is to apply weighted least squares (WLS) to principal component analysis regression. Unlike the traditional WLS, we introduce the locally weighted strategy into WLS and conclude the locally weighted PCA algorithms. For clarity, we briefly address the weighted PCA method, and then propose the locally weighted PCA in this section. After that, we address extreme cases.

A sequence of 3D skeletal motion data is usually represented in a matrix form. Let a sample of motion data be *A*_*i*_ ∈ *R*^*m*×3*n*^, *i* = 1‥*K*, where *m* is the number of frames, *n* is the number of markers, *K* is the number of training samples and *m* ≫ *n*. All the training samples may be stocked in a matrix $A={\left(A_{1}^{T},\ldots ,A_{K}^{T}\right)}^{T}$. Let the testing sample be *M* ∈ *R*^*m*×3*n*^, which contains *G* gaps. Each gap does not only refer to some missing marker and also indicate the beginning and ending time of missing this marker in a sample. We can apply these gaps to every training sample *A*_*i*_ so that the resulting training sample ${\tilde{A}}_{i}$ has the same gaps as the testing sample *M*. To emphasize every gap, we apply only one gap to all the training samples each time, ${\tilde{A}}_{i}^{g},i=1‥K$, where *g* denotes the index of gaps. As a result, we obtain a set of training samples for each full motion matrix *A*_*i*_, i.e., $\{A_{i},{\tilde{A}}_{i},{\tilde{A}}_{i}^{1},\ldots ,{\tilde{A}}_{i}^{G}\},i=1‥K$, and stack them by gaps into the individual gap-groups, ${\tilde{A}}^{g}=({\tilde{A}}_{1}^{g},\ldots ,{\tilde{A}}_{K}^{g}),g=1‥G$.

### Weighted PCA

Applying Singular Value Decomposition (SVD) to the training sample sets yields,

$$\begin{array}{c} \left\{ \begin{array}{c} A^{T}A=U\Sigma U^{T}\\ {\tilde{A}}^{g^{T}}{\tilde{A}}^{g}={\tilde{U}}^{g}{\tilde{\Sigma }}^{g}{\tilde{U}}^{g^{T}},g=1‥G \end{array}\right. \end{array}$$ (1)

where *U* and ${\tilde{U}}^{g}$ span the individual eigenspaces. In general, the principal component space is regarded as the sub-eigenspace spanned by the first *k* eigen vectors. (We still use *U* and ${\tilde{U}}^{g}$ to denote the principal component spaces in the following.) There exists a linear mapping *T*^*g*^ between the *U* and ${\tilde{U}}^{g}$. Thus we may assume that,

$$\begin{array}{c} A_{i}U\approx {\tilde{A}}_{i}^{g}{\tilde{U}}^{g}T^{g},i=1‥K,g=1‥G \end{array}$$ (2)

where *T*^*g*^ is a mapping of size *k* × *k* with regard to the *g*-th gap. The residual error is expressed as,

$$\begin{array}{c} B=\sum_{g=1}^{G}\sum_{i=1}^{K}(A_{i}-{\tilde{A}}_{i}^{g}{\tilde{U}}^{g}T^{g}U^{T}) \end{array}$$ (3)

Applying SVD to the residual *B*^*T*^*B* yields the eigenvalues {*δ*_*i*_}.

To weight the residual error, we can construct the weighted matrix *W* as a diagonal matrix with the diagonal of {1/*δ*_*i*_} through the training sample pairs. Usually, we need to set a threshold. When the *δ*_*i*_ < *threshold*, let *δ*_*i*_ = *threshold*. In weighted least squares, the *W* can overcome the issue of non-constant variance in samples.

We rewrite Eq 2 in a linear combination form as below,

$$\begin{array}{c} A_{i}\approx {\tilde{A}}_{i}(\sum_{g=1}^{G}\alpha_{g}{\tilde{U}}^{g}T^{g}U^{T}),i=1‥K \end{array}$$ (4)

where ${\tilde{A}}_{i}$ contains *G* gaps rather than one gap, and the regression coefficients *α*_*g*_ correspond to the gaps separately. To solve the unknown *α*, we employ the weighted least square method as follows,

$$\begin{array}{c} \underset{\alpha }{\text{min}}\sum_{i=1}^{K}\|(A_{i}-{\tilde{A}}_{i}(\sum_{g=1}^{G}\alpha_{g}{\tilde{U}}^{g}T^{g}U^{T}))W{\left(A_{i}-{\tilde{A}}_{i}\left(\sum_{g=1}^{G}\alpha_{g}{\tilde{U}}^{g}T^{g}U^{T}\right)\right)}^{T}\| \end{array}$$ (5)

It concludes the weighted PCA interpolation equation for the testing sample *M* as,

$$\begin{array}{c} M^{*}=M(\sum_{g=1}^{G}\alpha_{g}{\tilde{U}}^{g}T^{g}U^{T}) \end{array}$$ (6)

where M* denotes the reconstructed full motion matrix.

### Locally weighted PCA

Although the weighted least square method has good numerical stability and high computational efficiency, particularly it can deal with “outliers”, it still suffers the underfitting issue. When gaps stay at the areas where joints have the big fluctuations, it is hard for Eq 6 to improve the interpolation accuracy. The locally weighted strategy is to introduce a weighting mask over the motion matrix *M*, in which the entries far from the gaps will be given lower weights and the entries near to the gaps are given higher weights. This will taper the distance function to zero beyond a certain range. Mathematically, the mask will bring about some sparsity to covariance and result in an asymptotic optimal mean squared error. The mask is defined as,

$$\begin{array}{c} Q_{ij}^{g}=exp\{-\frac{dist^{2}\left(g,p_{ij}\right)}{\sigma^{2}}\},i=1‥m;j=1‥n;g=1‥G \end{array}$$ (7)

where *p* denotes an entry within a sample matrix, and *σ* denotes the window size of Gaussian function. *dist* denotes the distance from an entry *p* to the *g*th gap within a sample matrix, that is, a square root of the summation of the squared the makers’ spatial and temporal distances in a motion matrix. ′*i*′ indicates time dimension and hence it is used to compute the temporal distance. ′*j*′ stands for the markers. But, we compute the spatial distance between markers using the shortest path on a human skeleton model instead of the real distance between two markers here.

Moreover, for each gap, we may construct the individual mask *Q*^*g*^ and apply it to two sample sets *A* and ${\tilde{A}}^{g}$ respectively, which updates Eq 1 to yield the eigenspaces *U*^*g*^ and ${\tilde{U}}^{g}$ accordingly. We rewrite Eq 3 as,

$$\begin{array}{c} B=\sum_{g=1}^{G}(\sum_{i=1}^{K}(A_{i}-{\tilde{A}}_{i}{\tilde{U}}^{g}T^{g}U^{g^{T}}).*Q^{g}) \end{array}$$ (8)

where ′.*′ denotes the elementwise multiplication. The weighted matrix *W* can be constructed according to the eigenvalues of the covariance *B*^*T*^*B*. The regression coefficient *α* is solved by minimizing the residuals Eq 8 as,

$$\begin{array}{c} \begin{array}{c} \underset{\alpha }{\text{min}}\sum_{i=1}^{K}\|(A_{i}-{\tilde{A}}_{i}(\sum_{g=1}^{G}\alpha_{g}{\tilde{U}}^{g}T^{g}U^{g^{T}})).*\bar{Q}W\\ {\left(\left(A_{i}-{\tilde{A}}_{i}\left(\sum_{g=1}^{G}\alpha_{g}{\tilde{U}}^{g}T^{g}U^{g^{T}}\right)\right).*\bar{Q}\right)}^{T}\| \end{array} \end{array}$$ (9)

where $\bar{Q}=\sum_{j=1}^{G}Q^{j}$ and is quantified in [0‥1]. It concludes the interpolation equation for the testing sample *M* as,

$$\begin{array}{c} M^{*}=M(\sum_{g=1}^{G}\alpha_{g}{\tilde{U}}^{g}T^{g}U^{g^{T}}) \end{array}$$ (10)

Compared to the weighted PCA, the highlighted issue is the locally weighted *Q*, which is applied to the sample sets *A* and $\tilde{A}$ separately and results in the tapered covariance matrices. Essentially, the proposed locally weighted PCA is still a variant of weighed least square methods. Additionally, we only care about the interpolated items in M* of Eq 10. The others may be neglected.

#### Remark

The strategy of locally weighted mask *Q* is from the multivariate tapering approach, in which tapering, i.e., creating sparse approximate linear systems, has been shown to be an efficient tool in both the estimation and prediction settings [27]. In the missing marker interpolation scenario, we essentially construct the tapered covariances through a direct product of the presumed covariance function and a positive definite but compactly supported correlation function, i.e., the mask *Q*. Theoretically, multivariate tapering has shown asymptotic optimality for prediction, consistency, and asymptotic efficiency for estimation. In the application of motion data interpolation, our basic idea is to take the traditional weighted least square methods to suppress “outliers” and the covariance tapering technique to refine estimations. The “outliers” usually result in non-constant variances that are remedied by the traditional WLS. The sparsity of the tapered covariances can both remedy the underfitting issues and amend “outliers”. The covariance tapering method that we use is a bit different from the original version [27, 28], which applies the mask *Q* to the covariance to make it sparse. However it requires the tapered covariance maintains positive definiteness. In our implementation, the mask *Q* is applied directly to the sample data, i.e., Eq 8. Although the positive definiteness is guaranteed by the weight *W* in Eq 9, an issue is rising, i.e., is the resulting covariance Eq 9 sparse? In fact, Eq 9 is sparse, which may be simply explained as follows. Consider a sparse matrix *A* with mean zero. Let the covariance *C* = *AA*^*T*^. The diagonal entries of *C* indicate the squares of the norm of each row of *A*. The off diagonal entries indicate close and distant relationship among the rows of *A*. When two rows of *A* are similar, their off diagonal entry of *C* is high. Otherwise, the off diagonal entry is close to zero. If thresholding *C*, it can be noted that covariance is limited to a local neighborhood in *C*. Thus, *C* is sparse. Moreover, in terms of the multivariate tapering approach, the asymptotic mean squared error of the tapered covariance, i.e., Eq 9, can converge to the optimal error.

### Extreme case

#### —Missing whole frames

There are two extreme scenarios, missing whole frames and missing markers throughout. Due to sudden high frequency noise (or jitter) from the output, some frames are contaminated and have to be removed, which causes the issue of missing whole frames. Moreover, due to the detachment of markers throughout the MoCap session, this will result in the other issue of missing markers throughout. However, due to the de-meaning of the samples (i.e. removing the mean from the samples) in the pre-process stage, an accurate estimation of the mean is necessary to recover samples. In fact, it still remains challenging to estimate a proper mean in the scenario of missing markers throughout the MoCap session. In contrast, there is no such difficulty in the missing whole frames scenario. Our previous work [26] also shows the recovered samples are very sensitive to the estimated mean. We thus focus on the missing whole frames scenario in this paper.

Given an eigenspace *V* and the projection *a* of some sample *P* onto *V*, the sample can be expressed as *P* = *Va*. If dividing *V* into two parts, $V^{T}=\left(V_{1}^{T},V_{2}^{T}\right)$, the *P* may be reconstructed by them, P=(P1P2)=(V1aV2a), in which the same projection *a* is shared by these two parts of *V*. Thus, it is possible to reconstruct a part of *P* by the other one, $P_{2}=V_{2}{\left(V_{1}^{T}V_{1}\right)}^{-1}V_{1}^{T}P_{1}$. Moreover, if *P*_2_ contains one row vector while *P*_1_ containing all other vectors, it will effectively improve the estimation accuracy of *P*_2_. This is essentially a computation in terms of the correlation between frames. To this end, we introduce the Gram Matrix into Eq 1, which represents the inner product space, so that we can exploit the correlation between frames.

Let *A* = (*A*_1_, …, *A*_*K*_) and $\tilde{A}=\left({\tilde{A}}_{1},\ldots ,{\tilde{A}}_{K}\right)$, where each pair *A*_*i*_ and $\tilde{A_{i}}$ share the same shape but $\tilde{A_{i}}$ contains the missing frames. Let A˜i=(A˜i,1A˜i,2), where ${\tilde{A}}_{i,1}$ and ${\tilde{A}}_{i,2}$ correspond to the non-missing frame part and missing frame part respectively. *A*_*i*_ has the same partition, Ai=(Ai,1Ai,2). The eigenspaces can be constructed by SVD, *AA*^*T*^ = *V*Σ*V*^*T*^ and $\tilde{A}{\tilde{A}}^{T}=\tilde{V}\tilde{\Sigma }{\tilde{V}}^{T}$. There is a linear mapping *T* between *V* and $\tilde{V}$. We can divide *V* and $\tilde{V}$ according to the non-missing frame part and missing frame part,

$$\begin{array}{c} V^{T}=(V_{1}^{T},V_{2}^{T})=T^{T}{\tilde{V}}^{T}=(T^{T}{\tilde{V}}_{1}^{T},T^{T}{\tilde{V}}_{2}^{T}) \end{array}$$ (11)

and obtain the residual error as,

$$\begin{array}{c} B=A_{i,2}-{\tilde{V}}_{2}T{\left(T^{T}{\tilde{V}}_{1}^{T}{\tilde{V}}_{1}T\right)}^{-1}T^{T}{\tilde{V}}_{1}^{T}{\tilde{A}}_{i,1} \end{array}$$ (12)

Unlike Eq 3, the weighted matrix *W* is constructed by the eigenvalues of *BB*^*T*^, which unlikely results in zero eigenvalues due to a small number of missing frames in practice.

As the missing part on a sample is a set of whole frames, we may view each missing frame as a gap and set the missing frame number as *G* here. Without consideration of the markers’ spatial distances, we can remove the index ′*j*′ from the locally weighted mask Eq 7 and apply it to two sample sets *A* and $\tilde{A}$ to generate the individual eigenvectors *V*^*g*^, ${\tilde{V}}^{g}$ and the mapping *T*^*g*^ between them *g* = 1‥*G*, respectively. After that, we can solve the regression coefficients *α* through minimizing,

$$\begin{array}{c} \begin{array}{c} \underset{\alpha }{\text{min}}\sum_{i=1}^{K}\|{\left(A_{i,2}-\sum_{g=1}^{G}\alpha_{g}{\tilde{V}}_{2}^{g}H^{g}{\tilde{V}}_{1}^{g^{T}}A_{i,1}\right)}^{T}W(A_{i,2}-\sum_{g=1}^{G}\alpha_{g}{\tilde{V}}_{2}^{g}H^{g}{\tilde{V}}_{1}^{g^{T}}A_{i,1})\| \end{array} \end{array}$$ (13)

where $H^{g}=T^{g}{\left(T^{g^{T}}{\tilde{V}}^{g^{T}}{\tilde{V}}^{g}T^{g}\right)}^{-1}T^{g^{T}}$, *α* is a vector with *G* unknown regression parameters. We further conclude the locally weighted PCA regressor to recover the missing whole frames,

$$\begin{array}{c} M_{2}^{*}=\sum_{g=1}^{G}\alpha_{g}{\tilde{V}}_{2}^{g}H^{g}{\tilde{V}}_{1}^{g^{T}}M_{1} \end{array}$$ (14)

where *M*_1_ denotes the non-missing frame part of the testing sample *M* while $M_{2}^{*}$ for the recovery of missing frame part.

## Experiments

### Dataset and experiment settings

We conduct experiments on two famous datasets, i.e., the Motion Capture database HDM05 [29] and the CMU Motion Capture Database [30]. These two datasets contain up to 4000 motion frames with 41 markers. For convenience, we denote them as HDM and CMU respectively. In our experiments, two motion sequences are used for samples. Every sample is of 400 successive frames from the sequences.

**Fig 1:**
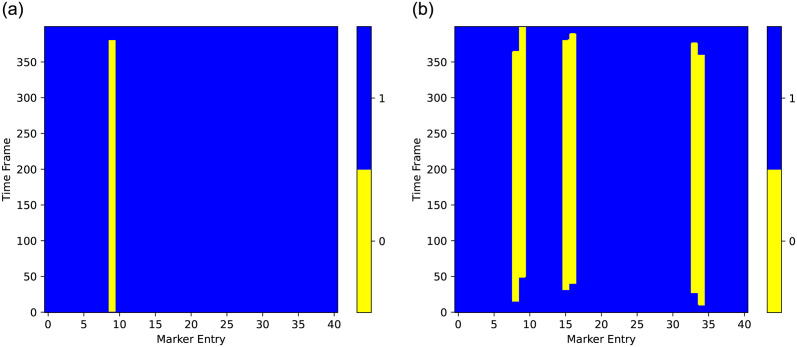
Examples of missing a single marker and multiple markers in a sample. YELLOW indicates gaps. (a) a single gap in HDM dataset. (b) multiple gaps in HDM dataset.

Consider two scenarios in our experiments, i.e., one missing marker and multiple missing markers in motion sequences. For the single missing marker case, we test each marker (or joint) with one random gap, in which the gap refers to one missing marker as adopted in [24]. Each sample is a sequence of 400 consecutive frames and each gap lasts 380 consecutive frames within it. For the multiple missing markers case, we produce three types of samples, including 3, 6 and 9 gaps randomly placed in a sample. Each gap occupies one marker. Fig 1 illustrates missing markers in a sequence sample. To reduce the influence of the randomness of generating samples, all the settings are executed 50 times. The final result is an average of 50 recovery errors. For the extreme case, we also produce four types of samples, including 3, 6, 9 and 12 consecutive whole frames separately as gaps. The gaps are randomly placed in a sample.

To compare with the state of the art methods, we focus on Probabilistic Model Averaging (PMA) [18] and two kinds of PCA-based reconstruction methods separately from Gloersen et al. [24] and our previous method [26]. Tits et al. [18] shows a good performance of their PMA against the other existing methods. Gloersen et al. [24] mentioned two methods, PCA_R1 and PCA_R2. Their experiments show that PCA_R2 outperforms PCA_R1. Our previous work [26] is closely related to [24] but the difference is to use training datasets. The other methods do not share their source codes for comparison such as Kalman Filter based gap filling algorithm [14]. Hence, we compare our proposed algorithms, Weighted PCA (denoted as WPCA) and Locally Weighted PCA (denoted as LWPCA), with PMA, PCA_R2 (denoted as PCA) and our previous method [26] in our tests.

The recovery error in our experiments is estimated by Mean Square Error (MSE) as follows,

$$\begin{array}{c} \epsilon =\frac{|\left(M^{*}-M_{grd}\right).*M_{ask}{|}_{Frob}^{2}}{m} \end{array}$$ (15)

where *M*_*ask*_ is a 0/1 matrix with the same shape as the sample M*, *M*_*grd*_ is the ground truth matrix and *m* is the number of missing entries in a testing sample matrix. The mean recovery error is to average the MSEs of 50 trials.

### Results

#### Missing single marker

Table 1 shows a comparison of our proposed algorithms (WPCA and LWPCA) and the other methods (PCA, PMA and [26]) on 5 markers(or joints). All the methods are carried out on the missing single marker setting through 50 trials for each marker respectively, that is, for each marker, a gap is randomly placed on its trajectory at each trial and the average values resulting from five methods are shown in Table 1. Thanks to the locally weighted mask strategy, our LWPCA outperforms the others. LWPCA’s results are noticeable better than those of the other methods though it can be noted that our algorithms’ performance(i.e., [26], WPCA and LWPCA) are very close on the CMU dataset compared to the HDM dataset. This is because the algorithm in [26] and WPCA are essentially least square method that can overcome noises but fails in “outliers”. LWPCA can overcome both noises and outliers. Our algorithms employ the training dataset (see Eq 1). Compared to the HDM training data, there are fewer outliers in the CMU training data. As a result, when performing our algorithms on two datasets, there are more prominent differences on the HDM data than on the CMU data.

**Table 1:** Mean recovery error for missing a single marker (The small value indicates small error according to Eq 15. The results of all joints in the S1 Appendix).

Joint index	CMU Dataset					HDM Dataset				
	PMA [18]	PCA [24]	[26]	WPCA	LWPCA	PMA [18]	PCA [24]	[26]	WPCA	LWPCA
9	4.0622	51.68518	2.1921	1.8936	**1.8708**	0.9753	0.4465	0.1536	**0.0534**	0.0560
14	17.11449	212.79873	8.1765	8.17647	**8.17622**	0.9827	2.4344	0.3410	**0.1756**	0.1877
21	34.7798	7.8435	**4.70464**	**4.70464**	4.70468	2.82383	25.06438	0.6902	0.54236	**0.52096**
30	28.88857	97.82413	**5.36026**	5.36027	5.36048	5.35315	11.13264	0.46882	0.32857	**0.27574**
33	**9.36036**	17.9440	9.98718	9.9872	9.98718	1.16865	13.66654	0.23407	0.11858	**0.11747**

**Fig 2:**
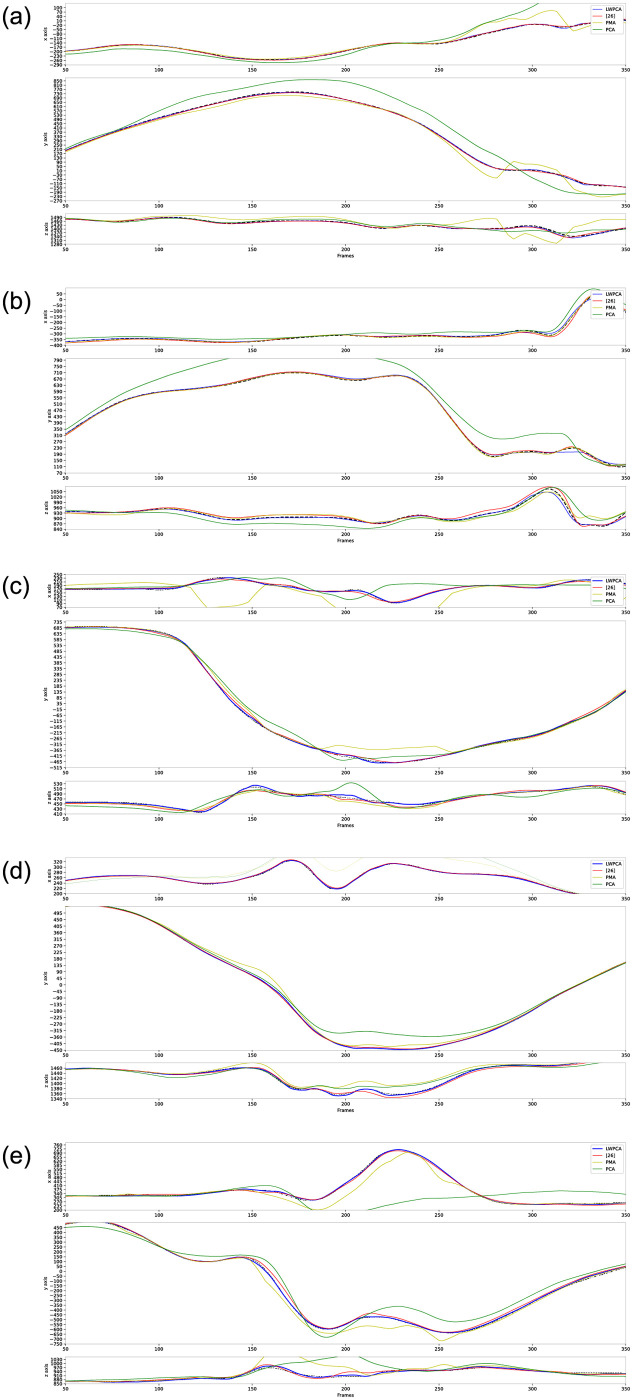
Comparison of the reconstruction for single missing gap at joints-9,14 on HDM data and joints-21,30,33 on CMU data respectively. Dotted lines represent the ground truth trajectories of joints. As WPCA is very close to LWPCA, it is not shown here.

Moreover it can be noted that the performance of our LWPCA on the joints-9,14 in the HDM group and the joints-21,30,33 in the CMU group are not best in Table 1 (for all the joints’ results, refer to the S1 Appendix). This is not strange. From a statistical perspective, the statistics of joints in Table 1 only show a kind of statistical average values that conceals numerical variance. The reconstruction of some joint’s trajectory can disclose the numerical variance of fitting curves and give us an insight to the methods. Fig 2 shows the trajectory reconstruction of these five joints with single missing marker. The ground truth trajectories of these five joints have big fluctuations, which tends to overfitting when interpolating gaps on them. Thus the stability of algorithms is prior to others here. We show the whole reconstructed trajectories instead of the interpolated gaps to highlight the numerical performance of our method-LWPCA, i.e., it can reach desired reconstruction accuracy. By contrast, the statistical averages in Table 1 cannot always accurately reveal the methods’ numerical performance. The important issue is that our algorithm-LWPCA demonstrates good numerical stability.

#### Missing multiple markers

**Fig 3:**
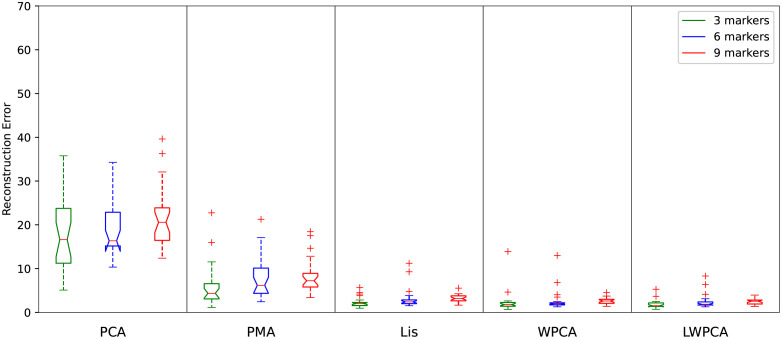
Boxplot of recovery errors for missing 3, 6, 9 markers in a testing sample using CMU dataset.

**Fig 4:**
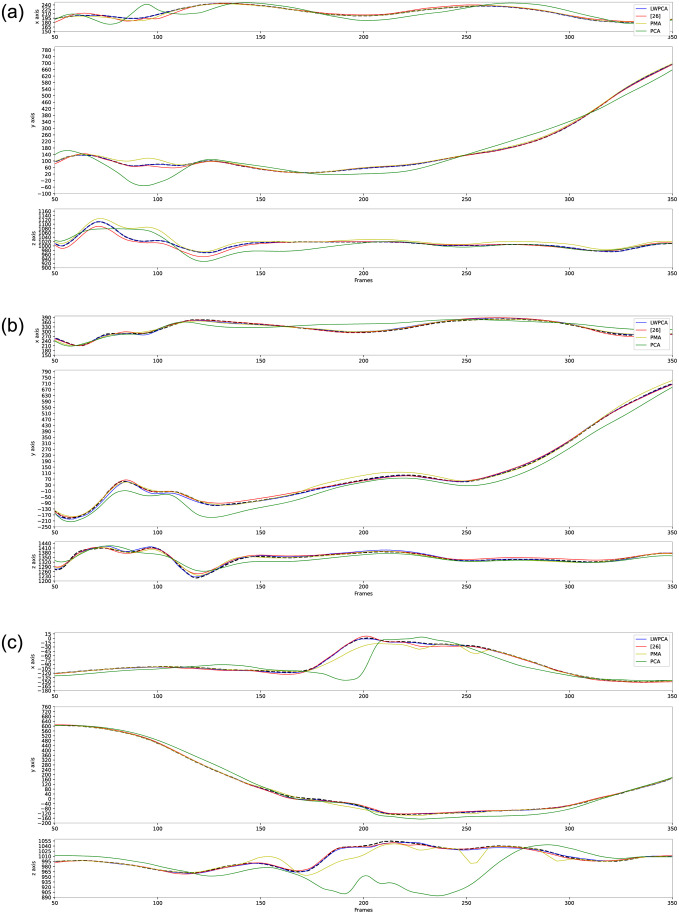
Reconstruction of the trajectory of joint-33 in the scenarios of missing 3,6 and 9 markers. Dotted lines represent the ground truth trajectories of the joint-33.

Table 2 shows the average recovery errors with increasing the number of missing markers in a testing sample. For a quantitative comparison, we give out the percentage of gaps over the whole sample in Table 2. Our LWPCA outperforms the others. Moreover, Fig 3 shows a boxplot of the recovery errors for missing 3, 6 and 9 markers in a testing sample. It can be noted that our algorithms ([26], WPCA and LWPCA) can gain a low variance of errors while the PCA and PMA methods suffer a big variance of errors. This means that our algorithms have a good numerical stability. It is also justified by Fig 4, which shows the joint-33’s trajectories when there are 3, 6 and 9 gaps in a sample respectively and the trajectory of joint-33 has gap all the time. Even if increasing the number of gaps, our LWPCA does not show evident degradation in performance. This isn’t indeed surprising since our algorithms employ training datasets.

**Table 2:** Mean recovery errors for missing multiple markers (the small figure indicates small error according to Eq 15).

Number of missing markers	CMU Dataset					HDM Dataset				
	PCA [24]	PMA [18]	[26]	WPCA	LWPCA	PCA [24]	PMA [18]	[26]	WPCA	LWPCA
3 markers (≈6% missing)	17.9949	5.7855	2.2986	2.3531	**1.8650**	0.4045	1.2494	0.2374	0.1156	**0.0996**
6 markers (≈13% missing)	19.1731	8.0698	3.0958	2.6342	**2.4313**	0.4387	1.4056	0.2598	0.1380	**0.1187**
9 markers (≈20% missing)	21.3471	8.1559	3.1711	2.5462	**2.4623**	0.5102	1.5147	0.2797	0.1383	**0.1350**

Additionally, PMA [18] employs a “Spacing Constraint” as its post-process in case of outliers, which can effectively enhance the algorithm robustness. We therefore use the PMA+constraints in our tests. For an intuitive comparison, we further perform our LWPCA on the same settings in [18] and compare the results with Table 2 of [18] in Table 3. Such low p-values indicate that the PMA suffers many “outliers” and heavily depends on the “Spacing Constraint” to suppress them. Furthermore, compared to Fig 3, it can be noted that our LWPCA evidently decreases the risk of “outliers”. Thus our LWPCA demonstrates good robustness.

**Table 3:** Mean recovery errors comparison with Table 2 of [18].

Dataset Name	PMA no constraint	PMA	p-value(PMA)	LWPCA
HDM_mm_01–02_03 (HDM1)	8.1	6.6	10e-5	**0.98**
HDM_mm_02–02_02 (HDM2)	4.8	4.4	0.46	**0.70**
HDM_mm_03–02_01 (HDM3)	5.5	5.2	0.07	**1.17**
HDM_mm_04–01_02 (HDM4)	8.5	7.4	0.08	**0.59**
HDM_bd_05–01_01 (HDM5)	4.5	3.0	0.001	**0.70**
85_02 (CMU1)	17.1	15.7	0.008	**3.28**
85_12 (CMU2)	14.4	13.5	10e-3	**2.32**
135_02 (CMU3)	10.5	8.4	10e-10	**1.95**

#### Extreme case–missing whole frames

We compare the LWPCA method with our previous method [26] in estimating the missed whole time frames in a sample since the other methods do not take into account such extreme cases. Table 4 shows the proposed LWPCA is evidently better than our previous method [26]. Moreover, comparing Table 2 with Table 1, it can be noted that the LWPCA performance is comparable. This justifies again that the proposed LWPCA has a good numerical stability.

**Table 4:** Mean recovery errors for missing whole frames.

Number of missing frames	CMU Dataset		HDM Dataset	
	[26]	LWPCA	[26]	LWPCA
3 frames (0.75% missing)	7.1326	**0.2181**	3.1761	**0.1788**
6 frames (1.5% missing)	12.9794	**0.4957**	10.1500	**0.2939**
9 frames (2.25% missing)	30.1606	**0.9831**	15.5814	**0.5337**
12 frames (3% missing)	53.9757	**1.1506**	28.4376	**0.9821**

## Conclusion

In this paper, we introduce the sparsity of observation data through the multivariate tapering approach [27] into traditional least square methods and develop it into the locally weighted least square scheme. It is the first least square method with the sparsity constraint and has a wide applications in prediction, estimation, regression analysis etc. To validate its numerical performance, we apply the proposed locally weighted PCA regressor (i.e., LWPCA Eq 10 to the “missing markers problem”. The experiment results show that the proposed LWPCA can reach high estimation accuracy and has a good numerical stability.

For motion data interpolation, our LWPCA demonstrates a good numerical performance though the extreme case-missing marker throughout is not taken into account. In fact, the distinct advantage of our methods (WPCA, LWPCA, [26]) is to employ training datasets. Using training datasets does not add computational burden since the complexity of matrix decomposition relies on the sample’s size rather than the amount of samples. Our LWPCA can work nearly in real-time. However, selecting training data still remains challenging. This will be our future work.

## Supporting information

S1 File Python codes of our algorithms. https://github.com/dangkh/LWPCA.
(ZIP)

S1 Appendix All joints’ results for missing a single joint. (PDF)

S1 Data (BST)
