# Supplementary material for: Locally weighted PCA regression to recover missing markers in human motion data
Source: PLoS One. 2022 Aug 8;17(8):e0272407. doi: 10.1371/journal.pone.0272407 (PMC9359544; doi:10.1371/journal.pone.0272407)
Supplement: S1 Appendix — (PDF) [file pone.0272407.s002.pdf]

Appendix

Table-Appx. Mean recovery errors for missing a single marker (including all the joints' results. The small value indicates small error according to Eq.??)

25emJoint index	**CMU Dataset**					**HDM Dataset**				
	PMA [?]	PCA [?]	[?]	WPCA	LWPCA	PMA [?]	PCA [?]	[?]	WPCA	LWPCA
1	2.04742	51.18811	1.08691	1.08691	**1.08583**	1.01656	10.84636	0.26246	0.02646	**0.0253**
2	1.53392	43.3709	1.11131	1.11131	**1.11012**	0.67489	10.65495	0.43486	0.03397	**0.03235**
3	32.09062	91.64048	4.25193	**4.25192**	4.25196	3.0738	15.91683	1.3076	0.99197	**0.91602**
4	18.39102	172.4829	5.13381	5.13381	**5.13265**	2.55804	28.5806	1.94452	0.91573	**0.91242**
5	21.30668	116.8931	6.95917	6.95917	**6.95807**	1.09197	28.28074	0.78152	0.57496	**0.57357**
6	21.06539	217.88403	17.53441	17.53443	**17.53335**	1.47354	28.48475	3.00966	2.32215	**1.45016**
7	9.43637	131.04378	9.08196	9.08214	**9.08194**	1.75427	34.41259	3.16077	1.68981	**1.67206**
8	9.03885	134.88964	7.83861	7.83859	**7.83732**	5.99677	47.45938	3.67788	3.24199	**2.8166**
9	4.0622	51.68518	2.1921	1.8936	**1.8708**	0.9753	0.4465	0.1536	**0.0534**	0.0560
10	8.95075	43.37945	2.59871	2.59872	**2.59771**	15.98887	32.95917	1.26536	1.06246	**1.019**
11	11.21124	106.67971	4.42166	**4.4215**	**4.4215**	11.32136	41.91879	1.4689	0.95774	**0.95589**
12	8.92721	86.60781	5.63325	5.63328	**5.63215**	6.08306	58.31204	1.21819	0.87148	**0.87048**
13	3.12877	34.29973	0.6377	**0.63768**	**0.63768**	2.28511	9.36921	0.29739	0.02922	**0.02815**
14	17.11449	212.79873	8.1765	8.17647	**8.17622**	0.9827	2.4344	0.3410	**0.1756**	0.1877
15	17.05293	82.64584	13.94923	**13.94917**	13.9488	11.70858	143.22681	4.86252	3.41924	**3.15646**
16	25.16466	132.89163	8.51334	8.51331	**8.51228**	20.74367	128.92855	6.42383	4.58531	**4.40026**
17	12.9567	30.41835	3.65216	3.65215	**3.65191**	0.71355	18.85588	0.26042	0.03038	**0.02936**
18	14.88697	35.11368	4.00824	4.00824	**4.0081**	0.91742	13.28004	0.26207	0.03566	**0.03456**
19	7.92528	24.75093	1.90446	1.90446	**1.90444**	2.60054	24.17103	0.22427	0.02364	**0.0226**
20	8.2317	29.22889	1.90622	1.90622	**1.90621**	0.93295	14.48337	0.2946	0.02729	**0.0262**
21	34.7798	7.8435	**4.70464**	**4.70464**	4.70468	2.82383	25.06438	0.6902	0.54236	**0.52096**
22	69.65764	72.20197	3.94174	3.94174	**3.94162**	3.5117	13.34765	0.39007	0.28642	**0.26122**
23	25.02785	147.58553	2.42624	2.42623	**2.42523**	5.11615	13.26878	0.32156	0.27659	**0.24561**
24	9.49028	32.28023	0.56181	0.56181	**0.56177**	10.96686	11.64703	0.62873	0.03871	**0.03727**
25	4.93326	160.71519	2.37433	2.37433	**2.37329**	4.67108	12.9328	0.21879	0.10222	**0.10118**
26	11.52563	187.17145	4.26122	4.26125	**4.26022**	10.1438	21.07924	0.13403	0.0611	**0.06008**
27	10.5262	179.90336	6.1801	**6.17994**	**6.17994**	5.20666	12.35446	0.14872	0.04706	**0.046**
28	6.9023	213.67059	1.7766	1.4929	**1.2338**	0.6319	0.2034	0.0802	0.0552	**0.0377**
29	31.09204	60.21932	4.43154	4.43154	**4.43046**	2.67347	9.90962	0.62971	0.50432	**0.4678**
30	28.88857	97.82413	**5.36026**	5.36027	5.36048	5.35315	11.13264	0.46882	0.32857	**0.27574**
31	14.03515	134.05522	3.39018	3.39016	**3.38909**	3.16673	12.55357	0.27979	0.22217	**0.21558**
32	6.34051	201.48603	6.02516	**6.0251**	**6.0251**	0.44164	7.94565	0.19116	0.13385	**0.13285**
33	**9.36036**	17.9440	9.98718	9.9872	9.98718	1.16865	13.66654	0.23407	0.11858	**0.11747**
34	18.25704	221.10989	12.88916	12.88928	**12.88883**	1.8375	13.29865	0.20431	0.08955	**0.08855**
35	14.45956	30.29359	**2.87638**	**2.87638**	**2.87638**	0.33944	11.2243	0.32973	0.03174	**0.03073**
36	15.37369	201.87589	9.19209	**9.19309**	**9.19309**	2.3299	11.95869	0.2238	0.07578	**0.07449**
37	11.92571	27.34079	6.90506	6.90506	**6.90486**	1.43483	12.7334	0.68402	0.06253	**0.05984**
38	51.50308	25.96686	4.04962	4.04961	**4.04859**	0.99813	8.18722	0.22994	0.02277	**0.02172**
39	34.35047	43.20834	5.49879	5.49913	**5.49878**	0.75835	8.98202	0.41785	0.03403	**0.03303**
40	8.29071	26.78191	7.91653	**7.91644**	**7.91644**	2.6283	12.91842	1.16027	0.87614	**0.83969**
41	33.98158	38.66417	4.84388	**4.84385**	**4.84385**	**1.45315**	13.90598	1.60272	1.94911	1.48716
